# Classification of arteriovenous fistula sounds using a convolutional block attention module and long short-term memory neural network

**DOI:** 10.3389/fphys.2024.1397317

**Published:** 2024-12-24

**Authors:** Jun Zhang, Rongxi Zhang, Xinming Shu, Hongtao Zhang

**Affiliations:** ^1^ School of Mechanical and Power Engineering, Zhengzhou University, Zhengzhou, China; ^2^ Department of Nephrology, People’s Hospital of Zhengzhou University, Zhengzhou, China

**Keywords:** CBAM-LSTM, feature fusion, AVF maturity classification, sound feature extraction, attention mechanism

## Abstract

The assessment of vascular accessibility in patients undergoing hemodialysis is predominantly reliant on manual inspection, a method that is associated with several limitations. In this study, we propose an alternative approach by recording the acoustic signals produced by the arteriovenous fistula (AVF) and employing deep learning techniques to analyze these sounds as an objective complement to traditional AVF evaluation methods. Auscultation sounds were collected from 800 patients, with each recording lasting between 24 and 30 s. Features were extracted by combining Mel-Frequency Cepstral Coefficients with Mel-Spectrogram data, generating a novel set of feature parameters. These parameters were subsequently used as input to a model that integrates the Convolutional Block Attention Module and a Long Short-Term Memory neural network, designed to classify the severity of AVF stenosis based on two sound categories (normal and abnormal). The experimental results demonstrate that the CBAM-LSTM model achieves an Area Under the Receiver Operating Characteristic curve of 99%, Precision of 99%, Recall of 97%, and F1 Score of 98%. Comparative analysis with other models, including VGG, Bi-LSTM, DenseNet121, and ResNet50, indicates that the proposed CBAM-LSTM model outperforms these alternatives in classifying AVF stenosis severity. These findings suggest the potential of the CBAM-LSTM model as a reliable tool for monitoring AVF maturation.

## 1 Introduction

The pathological voice is caused by abnormalities in the vocal system. The assessment of pathological voice in patients treated with blood dialysis mainly relies on manual examination (Sandoval et al., 2013), which is cumbersome, and often causes extreme discomfort. Moreover, the clinical analysis of the voice is often conducted based on subjective standards that are unsatisfactory. To overcome these difficulties, researchers have manually extracted features based on general classifiers (Gaussian mixture model, support vector machine, and hidden Markov model) to classify the pathological voice, including by integrating frequency cepstral coefficients (Barchiesi et al., 2015; Chachada and Kuo, 2014). Considering the superiority of the Convolutional Neural Network (CNN) over manual methods of feature extraction (Chachada and Kuo, 2014; Piczak, 2015; Pons and Serra, 2019; Salamon and Bello, 2017; Simonyan and Zisserman, 2014; Tokozume et al., 2017) even though it is primarily used for visual recognition, it has been successfully applied to analyze speech (Lee et al., 2009; Deng et al., 2013a; Abdel-Hamid et al., 2014; Sainath et al., 2013; Abdel-Hamid et al., 2012; Abdel-Hamid et al., 2013; Deng et al., 2013b) and music (Dieleman et al., 2011; Van den Oord et al., 2013). Among them, the convolutional attention mechanism is an improvement of the convolutional neural network. By combining space and channels, they are fused for multi-directional convolution to extract more important information, which enhances the network’s ability to extract effective information.

The traditional Recurrent Neural Network (RNN) is hindered by gradient-related issues, such as vanishing and exploding gradients, when dealing with long input sequences, leading to suboptimal performance. To resolve this challenge, Hochreiter introduced the Long Short-Term Memory (LSTM) model, which mitigates gradient issues by maintaining a continuous flow of error signals through its memory units. This ability to manage long-term dependencies makes LSTMs particularly effective for tasks involving sequences with significant delays between input and output (Schmidhuber, 1997). In the field of biomedicine, LSTM networks have been successfully applied to remove artifacts from EEG signals by leveraging their capacity to model long-range dependencies while filtering out sequential noise, thus preserving the integrity of the desired signal (Ghosh et al., 2023). Recent advancements have also proposed hybrid models combining CNNs with RNNs for applications such as Automatic Speech Recognition (ASR) (Sainath T. N. et al., 2015; Battenberg et al., 2016; Sainath T. et al., 2015) and music classification (Choi et al., 2017).

Neural networks learn the nonlinear mapping between input and output using training data, allowing them to generalize and achieve high classification accuracy on previously unseen data. Glangetas et al. (2021) proposed incorporating algorithms into digital stethoscopes, creating autonomous devices that can function as smartphone accessories, offering a more accessible diagnostic tool (Vasudevan et al., 2020). Early diagnosis and timely intervention, such as percutaneous transluminal angioplasty, are critical for managing AVF stenosis (Mccarley et al., 2001; Tessitore et al., 2004). Park et al. (2023). applied deep convolutional neural networks, specifically ResNet50, to predict AVF stenosis and its primary patency over 6 months using acoustic features like Mel spectrograms. Chen et al. (2021) studied the use of pulse radar sensors combined with machine learning classifiers to monitor and predict abnormalities in the flow function of AVFs. This method primarily relies on biometric signals captured by radar sensors and machine learning algorithms. Ota et al. (2020) employed a CNN-BiLSTM model to classify AVF sounds, achieving an accuracy of 70%–93% with an AUC of 0.75–0.92 after 100 training iterations. This method processes heartbeats' acoustic signals, classifying them by stenosis degree, contributing to more reliable AVF detection tools.

To further enhance classification performance, it is essential to convert sound signals into parameters that accurately describe their characteristics. For instance, Guo analyzed pathological sounds by extracting residual signal features, including the amplitude of the fundamental frequency, spectral flatness, and cepstral domain features, demonstrating their effectiveness in distinguishing between normal and pathological sounds when used in conjunction with traditional sound parameters (Guo et al., 2019). Similarly, Fang proposed a method that fuses pathological sound features by integrating Mel-frequency cepstral coefficients (MFCC) with non-linear vocal cord lesion features. This approach has shown promise in diagnosing and analyzing conditions such as coal workers' pneumoconiosis (Chunying, 2017). Ghaderpour et al. (2024) introduced a technique for computing confidence level surfaces for least-squares wavelet spectrograms, addressing the impact of measurement errors and missing data on spectral feature identification. This stochastic approach enhances the robustness of feature extraction, improving classification accuracy for AVF shunt sounds and potentially increasing diagnostic precision in AVF monitoring.

In this study, we present a method for classifying arteriovenous fistula sounds using a Convolutional Block Attention Module-Long Short-Term Memory (CBAM-LSTM) neural network. By integrating the Convolutional Block Attention Module with convolutional operations, the proposed model effectively captures key spatial and channel features from the AVF sound samples. The attention mechanism in CBAM improves the model’s focus on the most relevant aspects of the data, enabling enhanced feature extraction. Recent studies have demonstrated the successful application of CBAM in improving deep residual networks for recognizing complex patterns, such as symmetric and asymmetric human activities (Mekruksavanich and Jitpattanakul, 2024), further underscoring its adaptability. In our model, pooling operations are utilized for downsampling, reducing the dimensionality of the data and allowing the network to capture local structures and features within the audio signals more effectively. The Long Short-Term Memory layer is employed to process temporal data, enabling the model to capture long-term dependencies and handle sequential data while addressing the challenges of vanishing and exploding gradients. By combining these layers, the model can leverage both spatial and temporal information to enhance the classification of AVF sounds.

Additionally, we optimize the pathological sound features through a feature fusion algorithm that combines MFCCs and Mel spectrogram features, thereby improving the diversity of the audio data. The Mel spectrogram is widely adopted in audio analysis due to its effectiveness across various applications, and ongoing research continues to enhance its performance. For instance, Hu proposed a Mel spectrogram enhancement method based on Continuous Wavelet Transform, aimed at improving the clarity and quality of synthesized speech (Hu et al., 2024). The integration of CBAM, the LSTM network, and the fused feature parameters contributes to the high classification performance of pathological sounds. Comparative experiments demonstrate that the proposed CBAM-LSTM model, when combined with the fused feature parameters, outperforms other architectures such as VGGNet, Bi-LSTM, Densenet121, and ResNet50 in classifying AVF sound samples.

**FIGURE 1:**
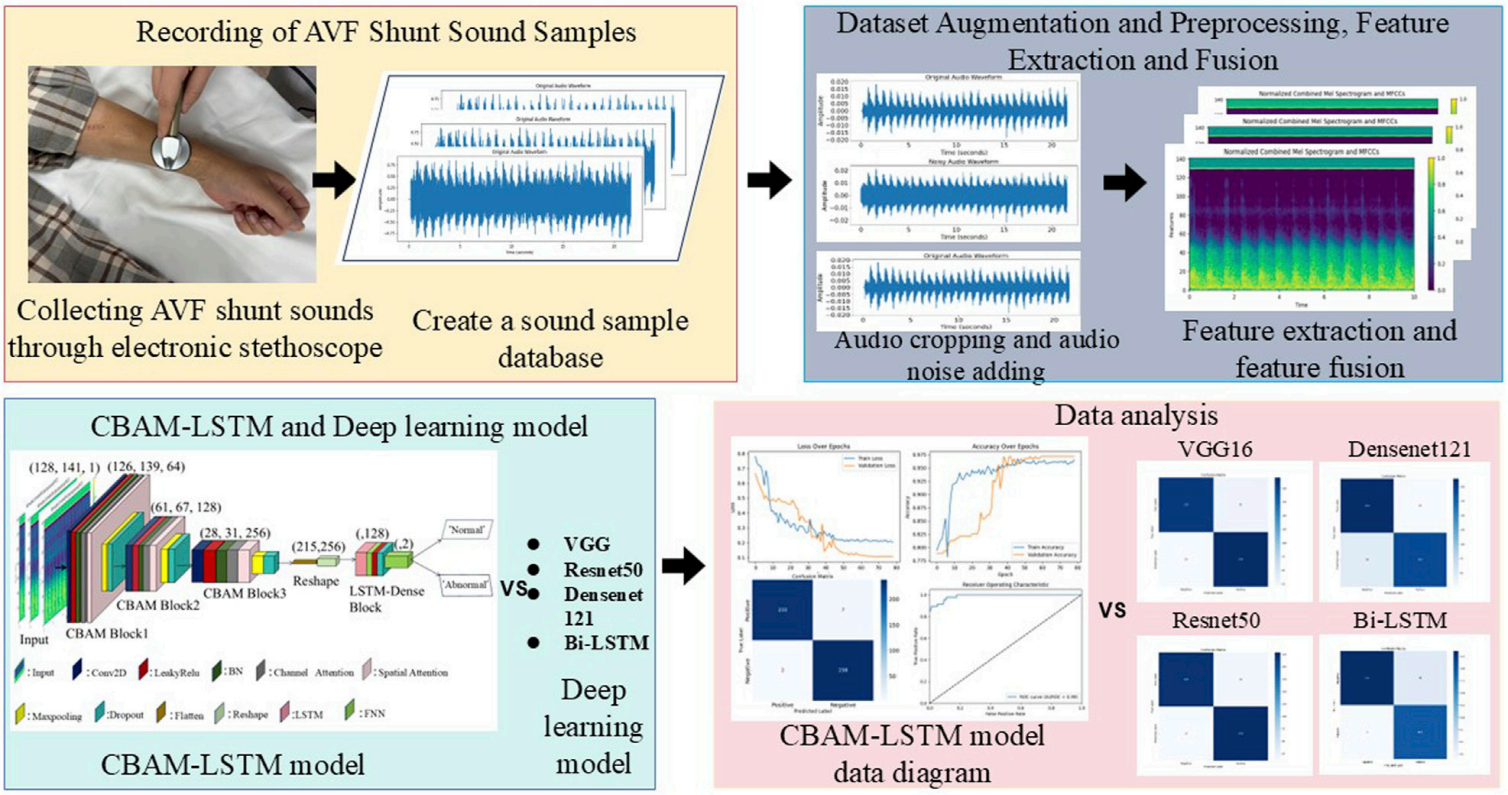
Flowchart of proposed method to classify sounds of the arteriovenous fistula based on the CBAM-LSTM neural network.

Our results have significant implications for the development of non-invasive diagnostic tools for monitoring vascular access in hemodialysis patients. Furthermore, the proposed method holds promise for broader applications, potentially extending to the classification of other types of biomedical signals. The framework of the proposed method is illustrated in Figure 1. The key contributions of this work are summarized as follows.

• Introduction of a Novel CBAM-LSTM Architecture: We propose a CBAM-LSTM neural network that effectively captures both spatial and temporal features of AVF sounds, thereby enhancing classification accuracy.
• Incorporation of an Attention Mechanism: The integration of CBAM with convolutional operations allows the model to focus on critical local spatial and channel features within AVF sound samples.
• Fusion of MFCCs and Mel Spectrogram Features: We apply a feature fusion algorithm to combine MFCCs and Mel spectrogram features, improving the representation of audio and boosting classification performance.
• Achievement of Superior Performance: Experimental results indicate that the proposed method outperforms existing models such as VGGNet, Bi-LSTM, Densenet121, and ResNet50, achieving state-of-the-art results in classifying pathological AVF sounds.

## 2 Data construction and feature fusion

### 2.1 AVF shunt sound database

The study utilized data from 800 dialysis patients who were hospitalized at the dialysis center of Henan Provincial People’s Hospital in China. These patients had undergone dialysis treatment through an arteriovenous fistula and maintained stable hemodynamics. The participants were hospitalized for various medical conditions and continued to receive maintenance dialysis. Data collection was conducted following the acquisition of informed consent from all participants. The characteristics of the participants are summarized in Table 1.

**TABLE 1:** Information on the dataset.

Feature	Value
Age	12–86
Gender	
Man	426
Woman	374
Have a history of AVF	591
Narrow area	
Above the anastomosis	576
Anastomosis channel opening	132
Head of the superior vena cava	48
Central vein	44

A wireless electronic stethoscope was positioned directly over the venous conduit at the anastomosis sites of 800 hemodialysis patients, and the sounds generated by the arteriovenous fistula (AVF) shunts were recorded for 24–30 s. These recordings were classified into two categories: “normal” and “abnormal.” In total, 800 AVF shunt sounds were captured, consisting of 600 normal and 200 abnormal samples, which were then stored as WAV audio files. Clinical assessments suggest that an arteriovenous fistula is considered stenotic when its diameter is less than 1.8 mm. Based on this criterion, sound samples obtained from fistulas with diameters smaller than 1.8 mm were classified as abnormal, while those from fistulas larger than 1.8 mm were categorized as normal.

### 2.2 Dataset augmentation and preprocessing

Training neural networks in a supervised manner faces challenges such as high computational workload and the need for labeled data. We evaluated the CBAM-LSTM network for classifying arteriovenous fistula sounds using 800 audio segments (24–30s each) at a 16 kHz sampling rate, balancing input quality and computational cost.

However, the training data available for AVF sounds was limited in both size and the number of abnormal samples, which created an imbalance between the normal and abnormal categories. This imbalance may hinder the neural network’s ability to generalize across varying scenarios and data variations, thereby restricting its performance in real-world applications. To address these challenges, we employed two strategies to augment the dataset of AVF sounds.

#### 2.2.1 Addition of audio noise

**FIGURE 2:**
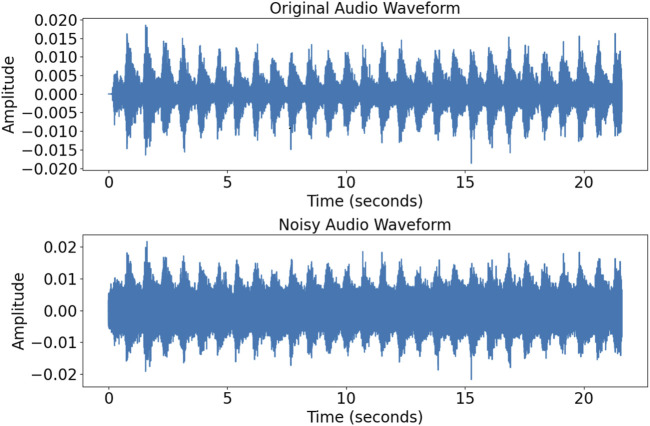
Comparison of signals of the time domain before and after the addition of noise. The top and bottom figures show signals of the time domain before and after the addition of noise, respectively.

To improve the model’s generalization, random white noise was added to the audio samples to simulate background interference. Figure 2 shows the original and noise-augmented waveforms, highlighting the increase in noise magnitude. This approach enhanced the model’s adaptability and robustness in diverse clinical conditions.

#### 2.2.2 Audio clipping

**FIGURE 3:**
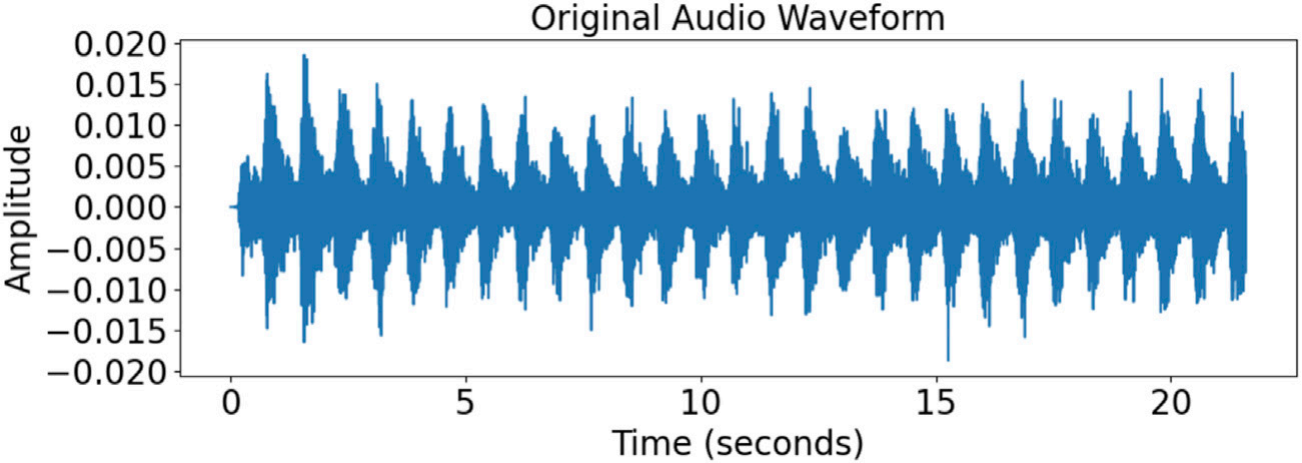
Sound samples cropped into audio clips with durations of 3–6 s and 18–21 s.

Figure 3 shows that we used the characteristics of the task and the dataset to extract audio segments with durations of 3–6 s and 18–21 s from the original 24–30 s long audio recordings to generate more training samples and increase the size of the dataset.

**FIGURE 4:**
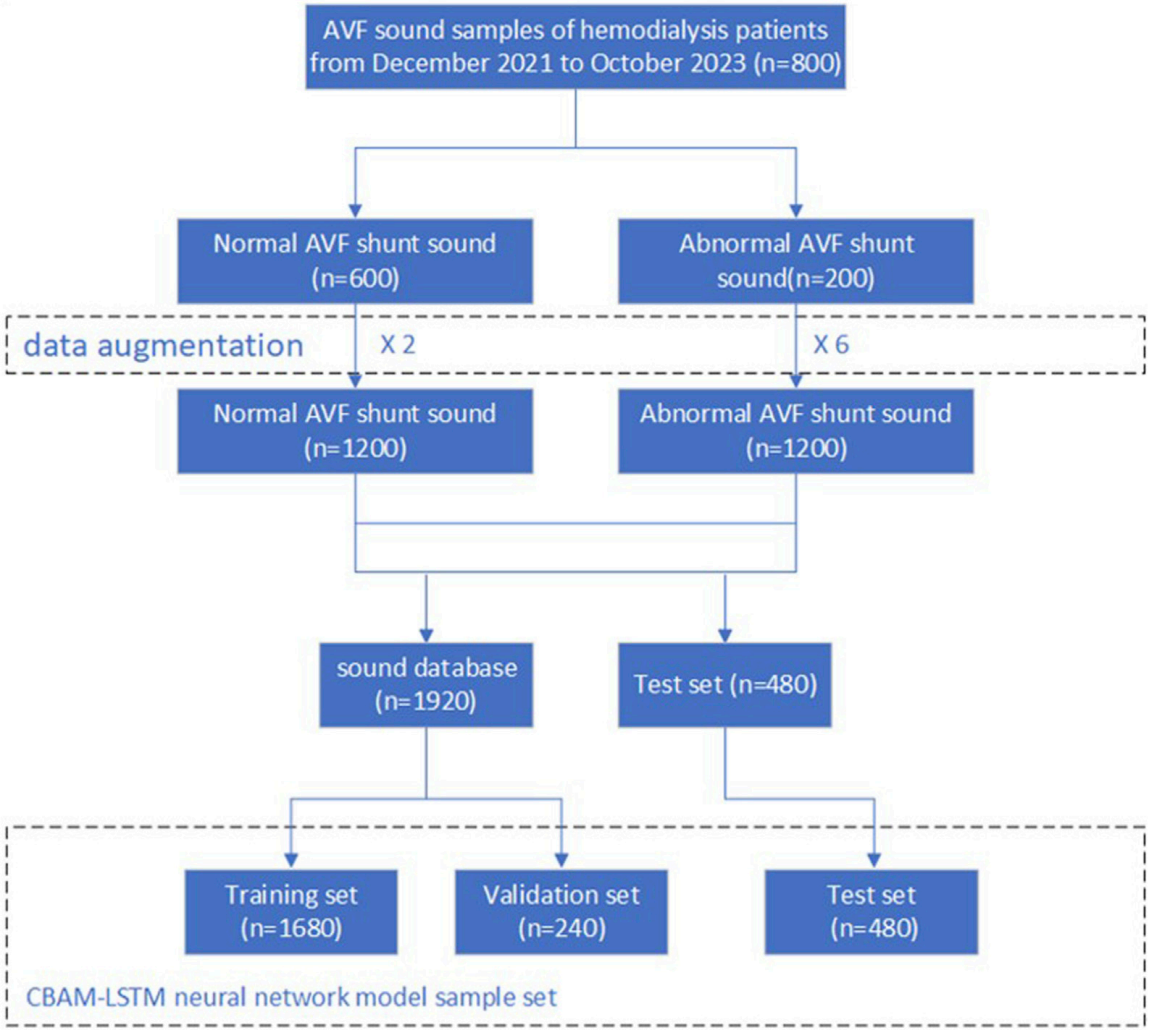
Flowchart of dataset expansion.

Following the augmentation of the dataset, it was randomly partitioned into training, validation, and test subsets, with allocation ratios of 70%, 10%, and 20%, respectively. This procedure is depicted in Figure 4.

The parameters of the dataset following its augmentation are presented in Table 2.

**TABLE 2:** Parameters of the dataset after its expansion.

Parameters	Value
Training Set	1,680
Test Set	480
Validation Set	240
Normal Samples	1,200
Anomalous Samples	1,200

We combined the Mel spectrogram with MFCCs to extract features from the audio data. Both the Mel spectrogram and MFCCs are widely recognized techniques for representing audio signals. Initially, we performed pre-processing on the audio signals to enhance their quality and prepare them for feature extraction. Specifically, we applied pre-emphasis to boost the high-frequency components, compensating for the natural attenuation that occurs in frequencies above 800 Hz. Pre-emphasis was achieved through the use of a specific transfer function, as represented by Equation 1.

$$H\left(Z\right)=1-\mu Z^{-1}$$ (1)

The aggravation coefficient μ in this paper is taken as 0.97. The result after the pre-emphasis treatment is shown in Equation 2.

$$y\left(n\right)=x\left(n\right)-0.97x\left(n-1\right)$$ (2)

**FIGURE 5:**
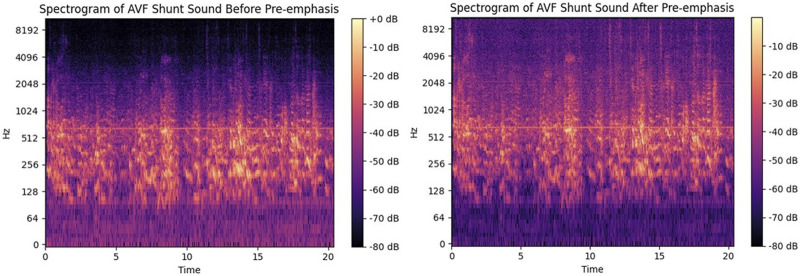
Spectrum before and after pre-emphasis. The left picture is the spectrum before pre-emphasis, and the right picture is the spectrum after pre-emphasis.

Where $x\left(n-1\right)$ represents the AVF blood flow acoustic input signal at the previous moment; $x\left(n\right)$ is the AVF blood flow acoustic input signal at the current moment; and $y\left(n\right)$ represents the output signal after pre-emphasis processing. Figure 5 presents the acoustic spectra both prior to and following pre-emphasis, emphasizing the alterations in the frequency distribution and the variation in the energy of the different frequency components.

### 2.3 Feature fusion algorithm

To generate a Mel spectrogram, the signal was initially segmented using a window of 25 m in length, with a step size of 10 m. The signal within each window was then transformed from the time domain to the frequency domain via a Fourier transform. Subsequently, the spectrum was mapped to the Mel scale using a Mel filter bank, producing the power spectrum. To standardize the dimensions of the samples, the resolution of the Mel spectrogram was adjusted to (128, 128).To acquire the MFCC, we set the same window length and step size as those used to acquire the Mel spectrogram to ensure signal stability in each frame. These signals were subsequently converted into the mel scale in the frequency domain and processed through a series of Mel filters to mimic the perception of different frequencies by the human ear. Following this, the log of power of the output of each filter was calculated and transformed into the cepstral domain by using the discrete cosine transform (DCT), yielding a set of MFCC features with dimensions of (128,13). Next, we use a feature fusion strategy to combine these two features. The process of feature fusion consists of three main steps.

#### 2.3.1 Normalisation

The features M of the Mel Spectrogram and the features C of the MFCC are normalised by the custom Normalize function, which scales the eigenvalues to the range [0,1], and for each element in the Mel Spectrogram features and the MFCC feature matrices, the normalisation process is shown in Equation 3.

$$\left\{\begin{array}{l} M_{norm,i,j}=\frac{M_{i,j}-\min\left(M_{:,j}\right)}{\max\left(M_{:,j}\right)-\min\left(M_{:,j}\right)}\\ C_{norm,i,j}=\frac{C_{i,j}-\min\left(C_{:,j}\right)}{\max\left(C_{:,j}\right)-\min\left(C_{:,j}\right)} \end{array}\right.$$ (3)

Where $M_{i,j}$ , ${M_{norm}}_{,i,j}$ represent the elements in row i and column j of the matrix before and after normalisation of the Mel spectrogram features, respectively; $C_{i,j}$ , ${C_{norm}}_{,i,j}$ represent the elements in row *i* and column *j* of the matrix before and after normalisation of the MFCC features, respectively; $M_{:,j}$ represents all the elements of the j-th column of the Mel spectrogram feature M ; $C_{:,j}$ represents all the elements of the j-th column of the MFCC feature C .

#### 2.3.2 Connected features

By splicing the Mel spectrum $M_{norm}$ with normalised dimension (128,128) and the MFCC feature $C_{norm}$ with normalised dimension (13,128) along the feature dimension, the *i*th row and j-th column of the fusion feature F can be defined by Equation 4.

$$\left\{\begin{array}{l} M_{norm,i,j},i\leq 128\\ C_{norm,i-128,j},i>128 \end{array}\right.$$ (4)

This process creates a new matrix F of dimension (141,128).

#### 2.3.3 Normalised convergence characteristics again

Normalise the fused features again: for the already fused feature vectors, the normalisation operation is performed again to ensure that each dimensional feature in the fused feature vectors is on the same scale, to prevent the new features from being fused to introduce a new bias, expressed as Equation 5:

$$F_{final,i,j}=\frac{F_{i,j}-\min\left(F_{:,j}\right)}{\max\left(F_{:,j}\right)-\min\left(F_{:,j}\right)}$$ (5)

Where $F_{final,i,j}$ is the feature matrix which is normalised again with the same dimensions as F . Finally, these MFCC features were combined with the features of the mel spectrogram and processed through a feature fusion algorithm to yield fused feature-related parameters with dimensions of (128,141). The data was then expanded to (128, 141, 1) to match the input requirements of the convolutional layer in the neural network model.

## 3 CBAM-LSTM neural network

We employed the CBAM-LSTM neural network model to analyze the acoustic signals of arteriovenous fistulas. This model integrates the Convolutional Block Attention Module for spatial feature extraction with the Long Short-Term Memory network for sequential data processing, providing a comprehensive framework for AVF acoustic analysis. The CBAM module preserves the gradient and important features from the convolutional layers by applying both max pooling and average pooling operations. These pooling operations are followed by a recalibration step, where feature weights are adjusted to emphasize critical regions, allowing the model to focus on the most informative areas in the data.

The process begins with the convolutional layer, where the output feature map $F\in R^{C\times H\times W}$ (with C , H , and W representing the channel, height, and width dimensions, respectively) is produced. Next, the CBAM applies the channel-wise attention mechanism by first computing the channel compression matrix $M_{S}\left(F\right)$ , followed by element-wise multiplication of F with the attention map, as shown in (Equation 6):

$$F_{1}=M_{S}\left(F\right)⊙F$$ (6)

where $M_{S}\left(F\right)\in R^{C\times 1\times 1}$ is the channel attention weight matrix, and ⊙ denotes element-wise multiplication. The spatial attention mechanism is then applied to the output $F_{1}$ ​, where the spatial compression matrix $M_{S}\left(F_{1}\right)$ is computed, followed by another element-wise multiplication, as shown in Equation 7:

$$F_{2}=M_{S}\left(F\right)⊙F_{1}$$ (7)

In this case, $M_{S}\left(F_{1}\right)\in R^{1\times H\times W}$ represents the spatial attention matrix, which is applied to adjust the feature map based on the most significant spatial regions. The spatial attention mechanism helps the model focus on critical spatial areas by reweighting the feature map according to the spatial attention map.

The final feature map $F_{2}$ ​ is thus a refined representation of the input features, where both spatial and channel attention mechanisms are applied to enhance the model’s focus on the most relevant information in the AVF acoustic signals. These processed features are then passed to the LSTM network, which models temporal dependencies and captures long-range correlations in the sequential data.

**FIGURE 6:**
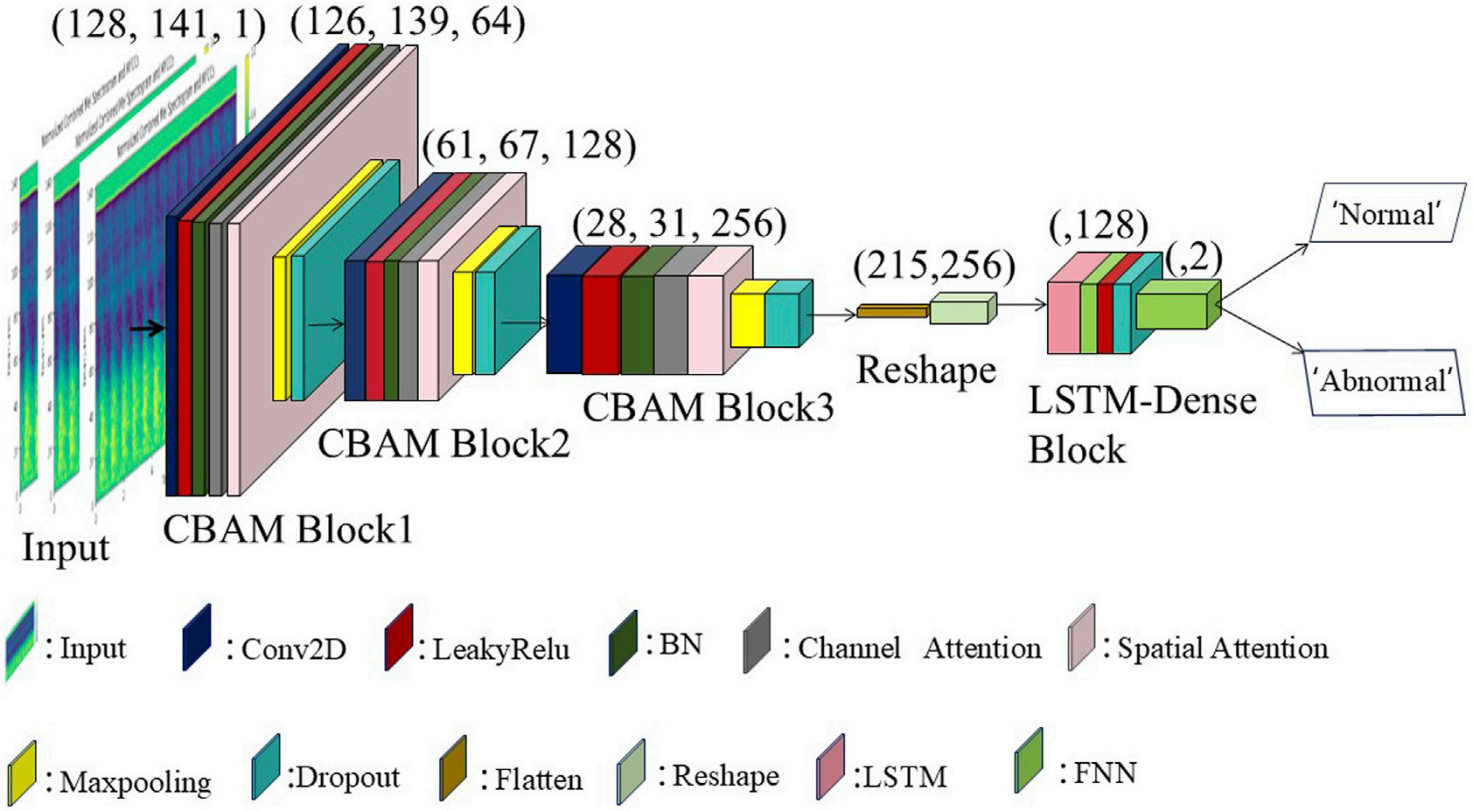
CBAM-LSTM neural network model.

The architecture of the proposed CBAM-LSTM model, shown in Figure 6, involves a series of multi-layered convolutional layers (Conv2D) that are responsible for extracting local spatial features from the acoustic signals. The first convolutional layer uses 64 filters, each of size 3 × 3, to extract the initial feature mappings, respectively, to refine and deepen the levels of the features. Each layer is closely followed by a LeakyReLU activation function that enables the network to model non-linear features while maintaining a portion of the negative gradient, thereby preventing zero activation values. Batch normalization layers are subsequently applied to each convolutional layer to normalize the data, and to enhance the efficiency of training and stabilize the performance of the network. Introducing channel attention modules and spatial attention modules after the BN (Batch Normalization) layer enhances the model’s ability to extract effective information.

Two-dimensional max-pooling layers then perform downsampling on the obtained feature mappings to reduce the computational load and prevent overfitting while retaining the most significant features. Dropout layers further enhance the capability of generalization of the model by randomly dropping some neuronal connections during training, thus reducing dependencies and improving the adaptability of the model to new data. The second and third parts of the model utilize convolutional layers with 128 and 256 filters, respectively, to further refine and deepen the hierarchical representation of features, while the remaining parts are identical to the first part.

Subsequent to the CBAM layers, the feature maps are passed through flattening and reshaping layers, preparing the data for processing by the Long Short-Term Memory network, which is responsible for capturing the temporal dynamics of the data. The LSTM layers are specifically designed to model the sequential patterns of the AVF sound signals over time, thereby addressing the long-term dependencies critical for accurately classifying the characteristics of AVF sounds.

Finally, two dense layers, which are processed through the LeakyReLU activation functions and the dropout layer, are responsible for generating the predictions of the network. The entire network is optimized by using a binary cross-entropy loss function as defined in Equation 8, with an Adam optimizer with a learning rate of 0.001 used during training. The batch size used for training was set for 300 rounds, with an early stopping mechanism incorporated into the callback functions.

$$L=-\frac{1}{N}\sum_{i=1}^{N}\left[y_{i}\log\left({\hat{y}}_{i}\right)+\left(1-y_{i}\right)\log\left(1-{\hat{y}}_{i}\right)\right]$$ (8)

where N represents the number of samples, $y_{i}$ is the actual label of the i th sample, which can be either zero or one, and $\hat{y_{i}}$ is the predicted label of the i th sample, signifying the probability predicted by the model of the sample belonging to category 1. The parameters of the CBAM-LSTM model are presented in Table 3.

**TABLE 3:** Training parameters of CBAM-LSTM neural network.

Parameters	Value
Learning rate	0.001
batch	64
Number of categories	2
Number of iterations	300

The CBAM-LSTM, model uses the above approach to integrate the capabilities of the CBAM, layers to extract spatial features with those of the LSTM, layers to process temporal data, thereby offering a powerful tool to analyze the acoustic signals of AVFs.

## 4 Experiments and analysis

### 4.1 Experimental platform and evaluation indicators

All training and testing was conducted on the same computer, which was equipped with a five vCPU Intel(R) Xeon(R) Platinum 8358P CPU @ 2.60 GHz, and an RTX 3090 GPU (24 GB). We used Python version 3.9 along with the Python libraries NumPy, matplotlib, scikit-learn, TensorFlow, and Keras.

We employed objective criteria to assess the performance of the CBAM-LSTM model. The evaluation metrics included the area under the receiver operating characteristic curve (AUROC), the confusion matrix, precision, recall, F-1 score, and accuracy. These metrics are defined by the formulas in Equations 9–12. Precision is the proportion of true positives among all instances predicted as positive by the model. Recall indicates the proportion of actual positive cases correctly identified by the model. The F-1 score represents the harmonic mean of precision and recall, while accuracy reflects the ratio of correctly predicted samples to the total number of samples.

$$Precision=\frac{TP}{TP+FP}$$ (9)

$$Recall=\frac{TP}{TP+FN}$$ (10)

$$F1Score=2\times \frac{Precision\times Recall}{Precision+Recall}$$ (11)

$$Accuracy=\frac{TP+TN}{TP+TN+FP+FN}$$ (12)

where TP , FP , TN , and FN respectively represent the number of samples correctly predicted as positive, the number of samples incorrectly predicted as positive, the number of samples correctly predicted as negative, and the number of samples incorrectly predicted as negative by the model.

### 4.2 Characteristic spectra of normal and abnormal AVF shunt sounds

The features of the spectrogram of AVF shunt sounds, both pre-percutaneous transluminal angioplasty (pre-PTA) and post-PTA, were qualitatively correlated with the degree of AVF stenosis.

**FIGURE 7:**
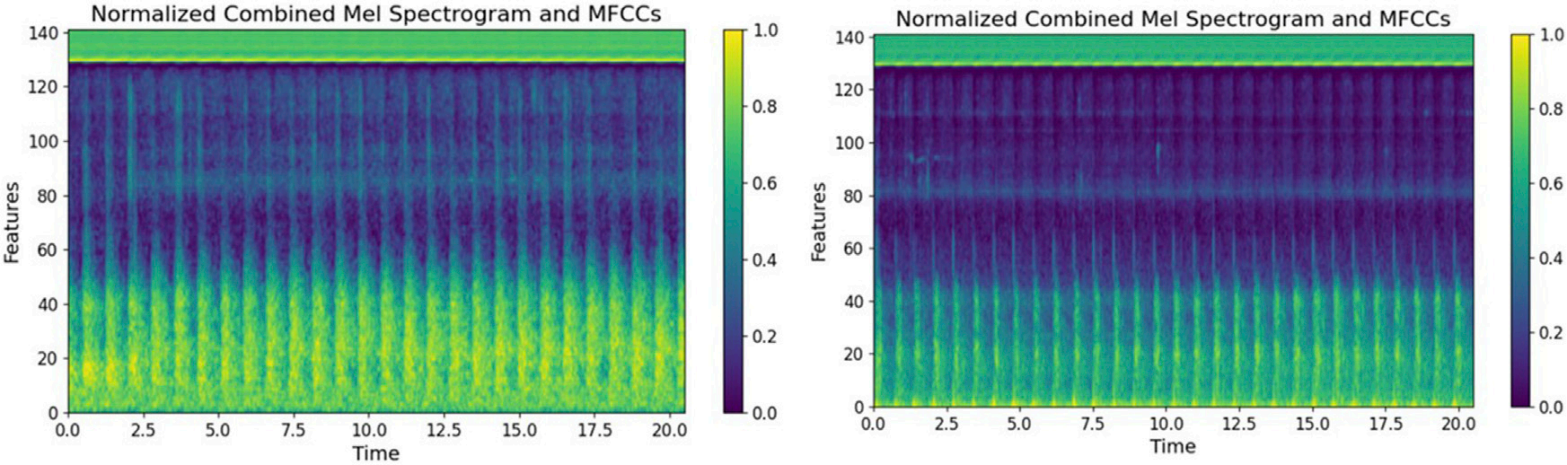
Parameter maps derived from the fusion of features. The left figure shows the characteristic spectrum of the parameter of normal AVF shunt sound, while the right figure shows the characteristic spectrum of the parameter of abnormal AVF shunt sound.

We collected shunt sounds from the AVFs and synthesized fused sound-related features by integrating the Mel spectrograms and the MFCCs by using a fusion algorithm. The horizontal axis in the fused feature parameter maps in Figure 7 represents time, measured in seconds, and the vertical axis denotes the feature dimensions. The brightness of the colors reflects the relative intensity of the energy at the respective time points and feature dimensions. The color bar represents normalized unit values, ranging from 0 to 1, without any physical units. It indicates the relative intensity of the feature values, where 0 corresponds to the minimum value, and one indicates the maximum value. We observed the features of energy distribution related to stenosis through a visual analysis of the fused feature-related parameter of the Mel spectrograms and MFCCs, and identified several key differences. The spectrograms of normal sound samples exhibited a relatively uniform energy distribution across the entire range of frequency, particularly in low-frequency areas, indicating that blood flow was not significantly obstructed. By contrast, the energy distribution of the fused feature-related parameter of the spectrogram of abnormal sound samples was scattered, and the high-energy area was not as prominent as that of the fused feature-related parameter of the spectrogram of normal sound samples. This means that the energy of abnormal AVF sounds in the entire spectrum was relatively low. Weak abnormal AVF sounds correspond to hemodynamic abnormalities caused by AVF stenosis.

To assess the performance variations among the fused feature parameter spectrogram, MFCC spectrogram, and Mel spectrogram, a random selection of 50 samples was made for comparison. The performance can be quantified using the following formulas for the Peak Signal-to-Noise Ratio (PSNR) and Structural Similarity Index (SSIM), as shown in Equation 13:

$$PSNR=10\cdot \log_{10}\left(\frac{MAX^{2}}{MSE}\right)$$ (13)

where MAX is the maximum possible pixel value of the image, and MSE is the mean squared error between the compared spectrograms, as shown in Equation 14:

$$SSIM\left(x,y\right)=\frac{\left(2\mu_{x}\mu_{y}+C_{1}\right)\left(2\sigma_{xy}+C_{2}\right)}{\left(\mu_{x}^{2}+\mu_{y}^{2}+C_{1}\right)\left(\sigma_{x}^{2}+\sigma_{y}^{2}+C_{2}\right)}$$ (14)

where $\mu_{x}$ ​ and $\mu_{y}$ ​ are the average intensities of the compared images, $\sigma_{x}^{2}$ and $\sigma_{y}^{2}$ ​ are their variances, $\sigma_{xy}$ ​ is the covariance, $C_{1}$ ​ and $C_{2}$ ​ are small constants to stabilize the division.

The average values of PSNR and SSIM are presented in Table 4. In this study, these metrics are used to investigate the differences between the Mel spectrograms of AVF blood flow in patients and those in healthy individuals, as well as to compare the performance of other spectrogram types.

**TABLE 4:** Differential indicator parameters.

Features	PSNR (dB)	SSIM
Mel spectrogram	15.1096	0.5256
MFCC	19.6950	0.7677
Fused Feature	8.58732	0.4344

A comparison of the data in Table 4 reveals that the fused feature parameter spectrogram displays the highest variability between normal and abnormal samples, with a PSNR value of 8.5873 dB and an SSIM value of 0.4344. This notable variability is advantageous for model training, as it facilitates the model’s ability to distinguish between normal and abnormal conditions. Consequently, when training the CBAM-LSTM model for AVF blood flow sound classification, the fusion of feature parameters emerges as the most effective approach to enhance classification accuracy.

### 4.3 Analysis based on evaluation indicators

**FIGURE 8:**
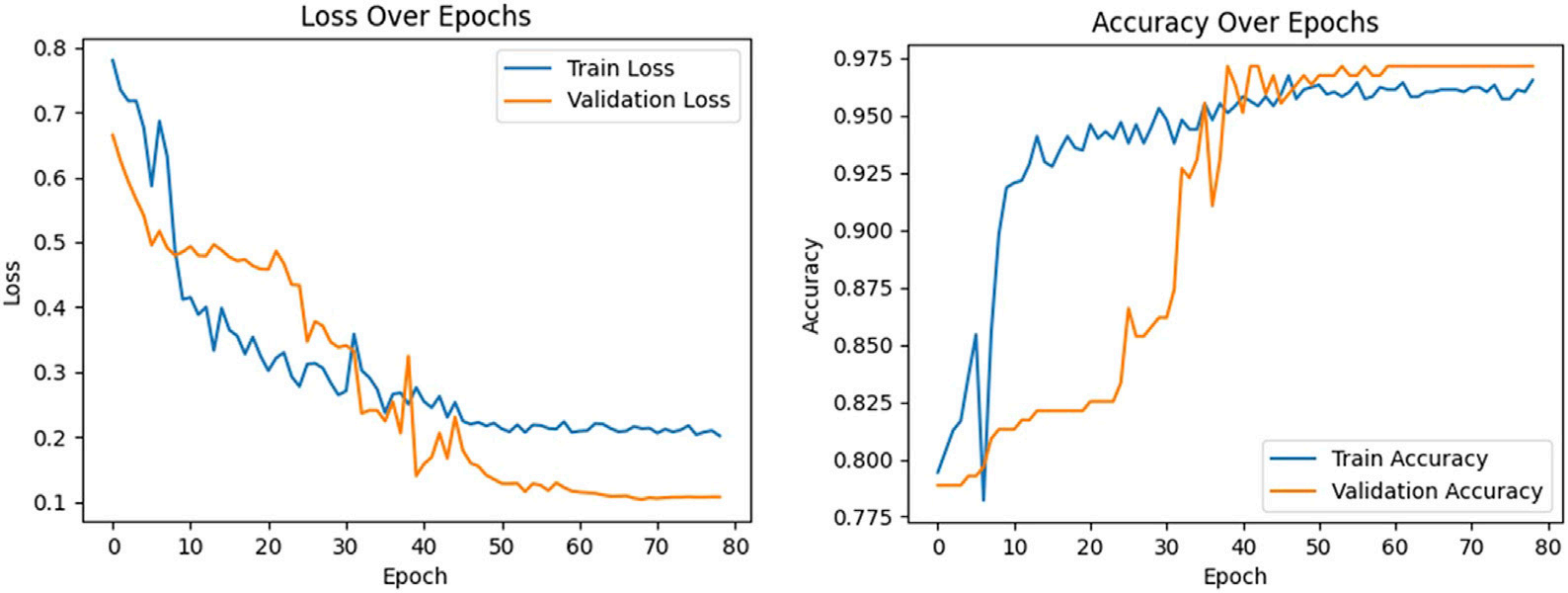
Curves of the loss function and accuracy of the proposed model.The left figure shows the training loss (blue curve) and verification loss (orange curve). The right figure shows the training accuracy (blue curve) and verification accuracy (orange curve).

A pivotal aspect of deep learning models is the iterative optimization of their parameters to minimize errors of classification. We used the loss function as a critical indicator that provides a quantifiable measure of the performance of the model in terms of the accuracy of classification. This metric plays a fundamental role in guiding the trajectory of optimization, and substantially influences the refinement of the model. Moreover, accuracy, which represents the ratio of correctly classified instances to the total number of instances, is an essential evaluative parameter. The curves of both the loss function and the accuracy are integral for assessing the performance of the model over its iterations, and are shown in Figure 8.

During the training of the model, a noticeable trend emerged as the number of epochs of training increased. Both the training loss (illustrated by the blue curve in Figure 8) and the validation loss (represented by the orange curve) exhibited a consistent decline. This trend continued until the 50th epoch, at which point both the training and validation losses reached a plateau, and stabilized at 0.21 and 0.11, respectively. Due to the early stopping mechanism added to the model, training was stopped after 80 epochs. The convergence and subsequent stabilization of these loss values were reflective of the effective learning and generalization of the model, with the close correlation between them suggesting a minimized risk of overfitting.

**FIGURE 9:**
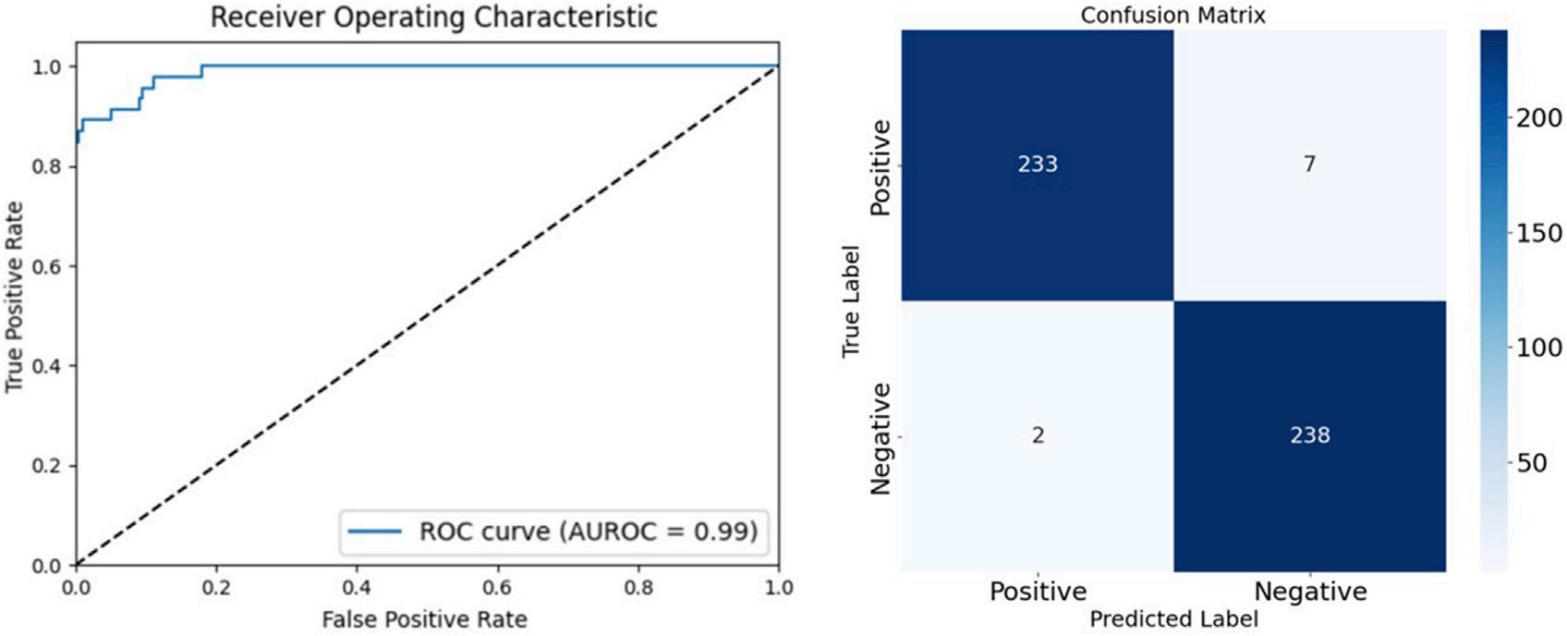
ROC curve and confusion matrix of CBAM-LSTM neural network in test set.

To thoroughly evaluate the performance of our arteriovenous fistula sound classification model based on the CBAM-LSTM neural network, we employed confusion matrices and ROC curves for result analysis. The confusion matrix is a widely used tool in medical image processing, offering a visual representation of the model’s classification performance, particularly in its ability to distinguish between positive and negative samples. The AUROC is utilized to assess the overall discriminative capacity of the model, serving as a robust measure for evaluating binary classification systems. A classification threshold of 0.5 was selected, as it is a standard value for binary classification tasks. Figure 9 presents the ROC curve and confusion matrix on the test set, providing insights into the model’s accuracy in distinguishing between normal and abnormal arteriovenous fistula sound samples.

The AUROC is a pivotal metric in this context. It provides a comprehensive measure of the discriminative capacity of the model, particularly in scenarios of binary classification. We opted for a classification threshold of 0.5, which is a standard benchmark in binary classification systems. The corresponding ROC curve is shown in Figure 8, and provides a visual representation of the capability of classification of our model under various thresholds.

It is evident from the ROC curve that the model had a high accuracy of recognition. The ROC curve was far from the diagonal line in the graph, which represents the performance of random guessing, while the curve of our model closely hugged the upper-left corner, indicating a near-perfect ability of classification. The AUROC value was 0.99, and approached the ideal value of one. This implies that the model can detect AVF stenosis with a very high true-positive rate while maintaining a low false-positive rate in medical diagnostic scenarios.

A detailed examination of the confusion matrix reveals that the model was highly precise. It correctly classified 238 instances as true negatives and erroneously categorized only two instances as false positives. This indicates a substantial reduction in the propensity of the model to commit type-I errors, and underscores its reliability in not misclassifying normal instances as anomalous. Moreover, the model was able to accurately identify 233 instances as true positives, with only seven instances incorrectly classified as false negatives. This reflects its commendable sensitivity in detecting abnormalities in the AVF and confirms its diagnostic accuracy. The results of the confusion matrix not only validate the robustness of our CBAM-LSTM model, but also show its potential for use in clinical scenarios for efficiently and accurately monitoring the AVF.

We also computed key statistical metrics to quantify the performance of our model, including the precision, recall, and F1-score. The model delivered a remarkable precision of 0.99, indicating its exceptional accuracy in correctly identifying positive instances. Its recall, or the true-positive rate, was 0.97, highlighting its efficiency in detecting and capturing positive samples. Its F1-score, a harmonized measure of the precision and recall, was an impressive 0.98. Collectively, these metrics demonstrate the reliability and effectiveness of our model in classifying sounds of the AVF. This verifies the effectiveness and reliability of the proposed CBAM-LSTM neural network model for the classification of AVF shunt sounds. Its evaluation metrics are presented in Table 5.

**TABLE 5:** Evaluation metrics of the performance of the proposed model.

Parameter	Value
Precision	0.99
Recall	0.97
F1 Score	0.98
Accuracy	0.97
Loss	0.11
AUROC	0.99

### 4.4 Comparison with other detection algorithms

We compared our CBAM-LSTM neural network model with three neural network-based approaches to learning: VGGNet, Bi-LSTM, Densenet121 and ResNet50. The diagnostic performance metrics of these methods are listed in Table 6.

**TABLE 6:** Evaluation indicators of four models.

Model	AUROC	Precision	Recall	F1 score
VGGNet	0.95	0.96	0.90	0.93
Bi-LSTM	0.97	0.97	0.93	0.95
DenseNet121	0.93	0.93	0.90	0.91
ResNet50	0.91	0.91	0.89	0.90
Ours	0.99	0.99	0.97	0.98

**FIGURE 10:**
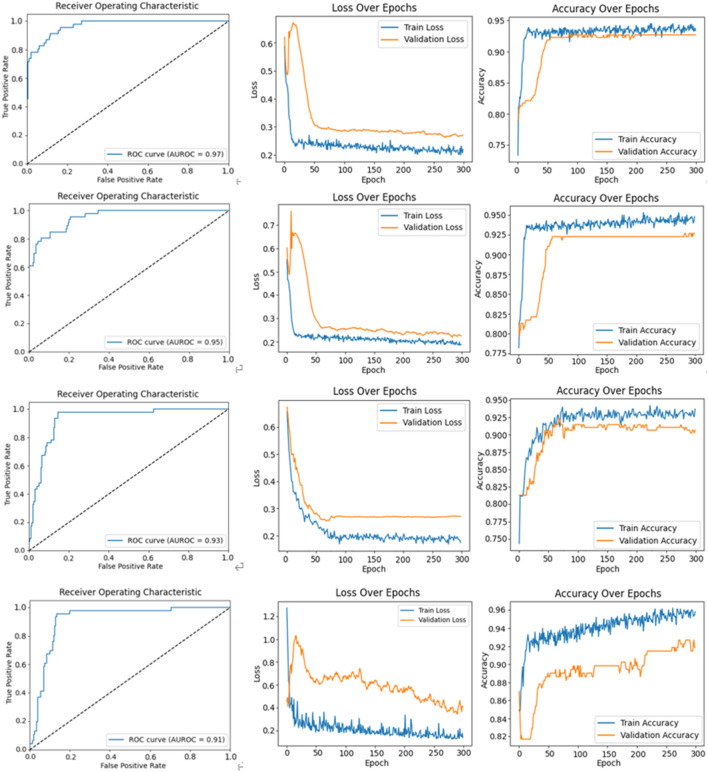
The figures illustrate the ROC curve, loss function curve, and accuracy curve for various models. The first row corresponds to the Bi-LSTM model, showing the ROC curve, loss function curve, and accuracy curve. The second row represents the same curves for the VGGNet model. The third row displays these curves for the DenseNet121 model, and the fourth row depicts the ROC curve, loss function curve, and accuracy curve for the ResNet50 model.

The ROC curves, loss curves, and accuracy curves for Bi-LSTM, VGGNet, Densenet121 and ResNet50 are shown in Figure 10. Our model demonstrated significant advantages over them across multiple performance metrics. It achieved an AUROC score of 99%, significantly surpassing the 97% recorded by Bi-LSTM, 95% by VGGNet, 93% by DenseNet121% and 91% by ResNet50. This indicates that our model had a higher accuracy and reliability in identifying the severity of AVF stenosis (i.e., normal vs abnormal).

Furthermore, our model achieved 99% in terms of precision and 97% in terms of recall. These results show its exceptional performance in terms of minimizing misclassification while ensuring that nearly all actually abnormal cases were correctly identified. It recorded an F1-score of 98%, which was noticeably superior to those of the Bi-LSTM (95%), VGGNet (93%), DenseNet121 (91%) and ResNet50 (90%). This demonstrates the superior capability of our model in balancing precision and recall.

**FIGURE 11:**
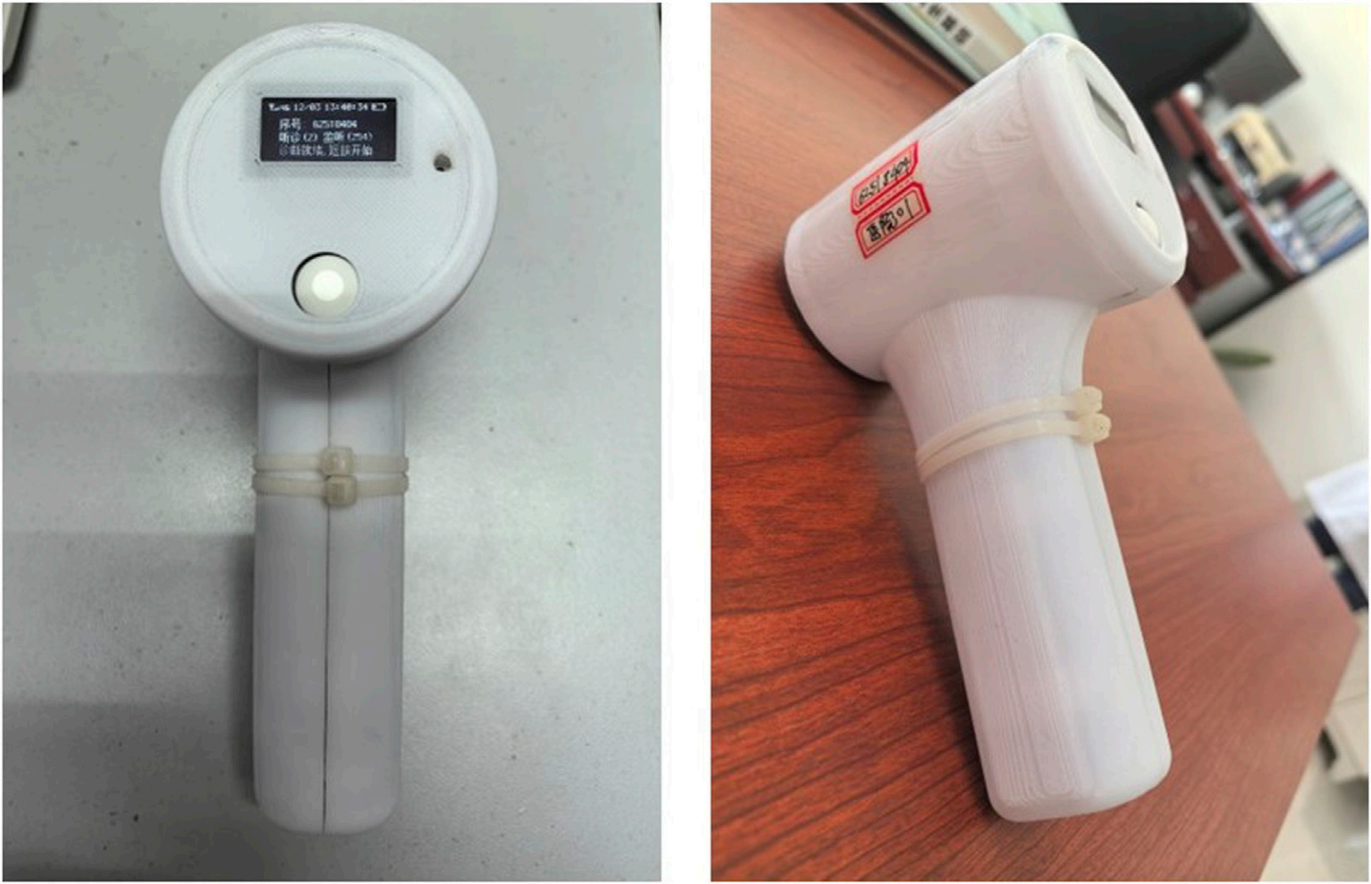
Figure a illustrates the front view of the device, while Figure b presents its side view.

In summary, our model not only offers superior diagnostic performance to that of the other models, but also ensures a more accurate and reliable monitoring of the AVF in patients undergoing hemodialysis. Finally, we developed an instrument that incorporates the CBAM-BiLSTM model, enabling it to perform non-contact assessment of arteriovenous fistula stenosis. The device is linked to the physician’s mobile phone via Bluetooth, with its parameters configured through a specialized application. The test results are subsequently transmitted to the server via the app.The device is shown in Figure 11.

## 5 Conclusion

Early detection and monitoring of arteriovenous fistula maturation are critical for patients undergoing hemodialysis, as early intervention can significantly improve clinical outcomes. In response to the need for non-invasive, accurate, and efficient diagnostic techniques, this study proposes an approach leveraging advanced neural network architectures to classify AVF shunt sounds with high precision.

We introduce a CBAM-LSTM model that combines the Convolutional Block Attention Module with Long Short-Term Memory networks to analyze acoustic signals from AVFs. By utilizing feature fusion techniques that combine Mel-frequency cepstral coefficients and Mel spectrograms, the CBAM-LSTM model enhances the ability to capture subtle differences in AVF sound data. This feature fusion improves the model’s diagnostic accuracy, making it better equipped to detect and classify AVF stenosis with high precision. This integration enhances the model’s performance, achieving an Area Under the Receiver Operating Characteristic Curve of 99%. Moreover, the CBAM-LSTM model outperforms alternative architectures, including VGGNet, Bi-LSTM, Densenet121, and ResNet50. These results highlight the promising potential of the CBAM-LSTM model for clinical applications in the monitoring of AVF maturation.

Future research should consider incorporating stochastic analysis techniques, such as the computation of confidence level surfaces for spectrograms, to further improve the robustness of feature extraction. Additionally, the exploration of multi-scale fusion methods combining ultrasound data with acoustic signals may enhance classification accuracy, facilitating more accurate diagnostics and personalized patient management.

## Data Availability

The raw data supporting the conclusions of this article will be made available by the authors, without undue reservation.
